# Immunological Effects of Protoscolex Antigen Injection on Hydatid Cyst Development in Mice

**DOI:** 10.12688/f1000research.174636.1

**Published:** 2026-01-29

**Authors:** Abdalwahab Bdewi Hussain, Anwar Khalil Ismael, Mohammed YI Al-Hamadani, Nuha Majeed Farhan

**Affiliations:** 1 Department of Microbiology, College of Medicine, University of Fallujah, Iraq; 2 Department of Pathological Analysis, College of Applied Science, University of Fallujah, Iraq; 3 Department of Biology, College of Education, University of Fallujah, Iraq; 4 Department of Pathological Analysis, College of Applied Science, University of Fallujah, Iraq

**Keywords:** hydatid disease, protoscolex, antibodies, Complement, PMNs, NBT.Mice, Echinococcus granulosus

## Abstract

**Background:**

Hydatid disease is a widespread disease that affects all human organs.

**Aims:**

This study aimed to evaluate antibodies (IgA, IgG, and IgM), complement proteins (C3 and C4), MIF of PMNs cells, reduction of nitro blue tetrazolium (NBT), and phagocytic activity against *Candida albicans.*

**Methodology:**

Hydatid cysts were collected from surgically injured patients at Fallujah Teaching Hospital, Twenty BALB/c mice were injected with Protoscolex antigen as treatment groups or phosphate-buffered saline (PBS) as control groups, at booster and challenge doses. Antibodies and complement concentrations were measured by enzyme-linked immunosorbent assay (ELISA), and the MIF of PMNs cells, reduction of NBT, and phagocytic activity against *Candida albicans* were measured to assess cellular activity.

**Results:**

The results showed significant differences in the levels of antibodies compared to the control group 21 days after injection of the protoscolex antigen at a concentration of 0.5 ml subcutaneously. There were also significant differences between the treatment and control group in the levels of C3 and C4. NBT dye reduction and phagocytosis of *Candida albicans* by PMNs showed significant differences compared to the control group. The results also showed a significant decrease in PMN viability in the treatment group. An increase in neutrophil migration diameter was observed. The results showed a 50.4% decrease in the infection level in the treatments injected with the antigen, and the average diameter of the hydatid cysts decreased compared with the control group.

**Conclusions:**

The results indicate that Protoscolex antigen activates the immune response and reduces severity of hydatid disease. This highlights its potential as a promising candidate for developing a vaccine or preventive strategy against hydatid disease.

## Introduction

Hydatid cysts are a common zoonotic disease caused by the eggs of the adult worm, *Echinococcus granulosus.* These eggs hatch into larvae in the intestine, penetrate the intestine, and migrate through the bloodstream to the liver, lungs, brain, and all parts of the body, forming hydatid cysts. This disease remains asymptomatic for approximately four years. ^1, 2^ Hydatid cysts can cause various symptoms depending on their size and location. ^3^ Humans can contract *E. granulosus* from the feces of infected dogs. Parasite eggs are consumed by humans through tainted food or drinks. After consumption, these eggs hatch into intestinal larvae that have the potential to grow into cysts. ^4^ The disease manifests as multiple cysts that affect various organs, particularly the liver and lungs. ^5^ The host’s immune system interacts with Taeniid cestode antigens (produced from juvenile intestinal stages, adult worms, or oncospheres) and may produce specific antibodies. It is widely acknowledged that the immune response has no effect on *Echinoccocus* species while their developing. ^6^ Also, animals experimentally infected with *E. granulosus* eggs or oncospheres have been shown to differ among mouse strains. Additionally, *in vitro* tests have demonstrated that neutrophils may destroy *E. granulosus* oncospheres when paired with antibodies, indicating a potential function of antibody-dependent cell-mediated cytotoxicity responses. Cell-mediated immunity to parasites is significantly activated in the early stages of the disease. ^7^ It is suggested that this protoscolex antigen is a good candidate for protection against hydatid cysts. Infection of BALB/c mice with *E. granulosus* constitutes a model for the study of secondary hydatidosis and the associated immune response in immunization and infection trials. ^8^ Given the widespread prevalence of the disease in Iraq, Turkey, Iran, Saudi Arabia, Egypt, and Kuwait. ^9, 10^ We conducted this experiment to determine the effect of injecting Protoscolex antigens into isolated human hydatid cysts on immune factors and the possibility of obtaining a vaccine against infection.

## Materials and methods

### Collection of the hydatid cyst for the injured people

Hydatid cysts were isolated from infected individuals after surgical procedures at Al-Fallujah Teaching Hospital.

### Experimental design and animals

Twenty BALB/c mice were obtained from the Drug Control Center of Iraqi Ministry of Health and Environment. Male and female mice aged 5-6 weeks, it weighed 15–29 g each. All animal procedures, including anesthesia and euthanasia, were conducted in accordance with the guidelines of the AVMA for the Euthanasia of Animals and were approved by the Institutional Ethics Committee at the College of Medicine, University of Fallujah (Approval No.: UOF.MED.05-240911001). Animals were euthanized under deep general anesthesia induced by isoflurane inhalation, followed by cardiac puncture for blood collection as a terminal procedure. These mice were divided as follows: the first group received five mice injected subcutaneously with 0.5 ml protoscolex antigens then on 14 day boosted with the same antigen and then dissection on 21 day to evaluate antibodies, complement proteins, the MIF of PMNs cells, the reduction of the nitro blue tetrazolium (NBT), and the phagocytic activity against *Candida albicans.* The second group was injected with 0.5 ml phosphate buffer solution (PBs) then on 14 day was boosted with the same concentration of PBs and then dissection on 21 day and was considered a control group. The third group received protoscolex antigen (0.5 ml protoscolex) subcutaneously. Fourteen days later, they received a second dose at the same concentration. A challenge dose was administered 21 days later and protoscolex heads were injected into the liver, and the mice were maintained for six months. The antigens were then examined to determine the extent of protection against hydatid cysts and the levels of immunoglobulins. The fourth group was injected with 0.5 ml PBs, and the second dose was administered after 14 days. On day 21, it was injected with protoscolex heads in the liver and was considered the control group, as shown in the following Table:

**Table:** Experimental Design Table.

Group	Number of Mice	Day 1	Day 14	Day 21	After 6 Months
**1**	5	Injected with Protoscolex antigen (0.5 ml)	Boosted with the same antigen (0.5 ml)	Dissection and measurement of tests	—
**2**	5	Injected with Phosphate buffer (PBS) (0.5 ml)– Control group	Boosted with the same buffer (0.5 ml)	Dissection and measurement of tests	_
**3**	5	Injected with Protoscolex antigen (0.5 ml)	Boosted with the same antigen (0.5 ml)	Injected with live Protoscolex in liver (challenge dose) (0.5 ml)	Dissection and measurement of tests
**4**	5	Injected with Phosphate buffer (PBS) (0.5 ml)– Control group	Boosted with the same buffer (0.5 ml)	Injected with live Protoscolex in liver (challenge dose) (0.5 ml)	Dissection and measurement of tests

### Preparation methods

The Taherkhani and Rogan ^11^ method was adopted for the preparation of the protoscolex antigen, in which an ultrasonic vibrator at 120,000 rpm for 5 min was used to break down the protoscolex. Total protein was measured according to the manufacturer’s instructions (Biolab) and the total protein amount was 3.864 mg/100 ml. The protoscolex was obtained from the germ layer of the hydatid cyst. Nitroblue tetrazolium (NBT) dye and eosin stain dye were prepared according to the method described by Metcalf et al. ^12^ method. In addition, Primary head samples were collected using the Symth method. ^13^

### Antibodies and complement test

Enzyme-Linked Immunosorbent Assay (ELISA) was used to determine the concentrations of the antibodies (IgG, IgM, and IgG) and complement (C3 and C4) using an ELISA kit from the French company (Sanofi).

### Statistics method

IBM SPSS statistics version 26.0. was used in this study. Data are expressed as the mean ± standard deviation (SD), by used t-test at a probability level of 0.05 (p < 0.05).

## Results

Table 1 shows the effect of protoscolex antigen injections on antibody concentrations. The results showed that antigen injection had a significant effect on all types of antibodies studied. The IgG, IgA and IgM antibody level reached (1435±496.2, 330.2±119.5 and 154.5±4.07) mg/100 ml, respectively, compared to control group (1012±1.8, 330.1±137.4 ^b^ and 111.8±57.8) mg/100 ml, respectively.

**Table 1.** The effect of subcutaneous injection of protoscolex antigens at a concentration of 0.5 ml on the concentration of antibodies after 21 day (mg/100 ml).

Concentration	IgG [mg/100ml]	IgA [mg/100ml]	IgM [mg/100ml]
	Mean±SD	Mean±SD	Mean±SD
0.5	1435±496.2 ^a^	330.2±119.5 ^a^	154.5±4.07 ^a^
PBs 0.5	1012±1.8 ^b^	330.1±137.4 ^b^	111.8±57.8 ^b^

Different characters indicate the presence of a significant difference (a,b) between the parameters at a level < 0.05.

The results of complement showed that, the C3 and C4 levels showed a significant decrease (125±9.6 and 21.3±5.4) mg/100 ml, respectively, compared to control group (165.8±36.2 and 54.7±3.1) mg/100 ml respectively, as shown in Table 2.

**Table 2.** The effect of subcutaneous injection of protoscolex antigens at a concentration of 0.5 ml on the concentration of complements after 21 day (mg/100 ml).

Concentration	C3 [mg/100 ml]	C4 [mg/100 ml]
	Mean±SD	Mean±SD
0.5	125.4±9.6 ^a^	21.3±5.4 ^a^
PBs	165.8±36.2 ^b^	54.7±3.1 ^b^

Different characters indicate the presence of a significant difference (a,b) between the parameters at a level < 0.05.

The results of the NBT dye reduction experiment for PMNs cells showed no significant difference between the results, as the percentage of cells reducing the dye reached (16.6±0.8) compared to the control group (13.6±0.9), as shown in Table 3.

**Table 3.** The effect of protoscolex injection subcutaneous concentration 0.5ml on percentage of reduction of NBT dye in the white mice after 21 day.

Concentration	NBT (%)
	Mean±SD
0.5	16.6±0.8 ^a^
PBs	13.6±0.9 ^a^

Different characters indicate the presence of a significant difference (a,b) between the parameters at a level < 0.05.

Regarding the viability of PMNs cells, the results showed a significant decrease in the percentage of cells in mice injected with antigens, reaching (84.9±2.1) compared to the control group (91.6±1.3), as shown in Table 4.

**Table 4.** The effect of 0.5 ml subcutaneous injection of protoscolex antigens on PMNs cell viability (%) after 21 day.

Concentration	PMNs cells %
	Mean±SD
0.5	84.9±2.1 ^a^
PBs	91.6±1.3 ^b^

Different characters indicate the presence of a significant difference (a,b) between the parameters at a level < 0.05.

As for the diameter of the migration circle of neutrophils, the results showed a significant increase in the antigen-treated animals (28.1±1.4 mm) compared to the control group (21.9±1.9 mm), as shown in Table 5.

**Table 5.** The effect of subcutaneous injection of protoscolex antigens on MIF at a concentration of 0.5 ml after 21 day.

Concentration	Migration of PMNs/mm	MIF
0.5	28.1±1.4 ^a^	1.6
PBs	21.9±1.9 ^b^	1

Different characters indicate the presence of a significant difference (a,b) between the parameters at a level < 0.05.

Regarding the effect of antigen injection on phagocytosis of *Candida albicans* yeast, the results showed a significant decrease in the number of PMNs cells treated with the antigen. The highest percentage of decrease occurred within 30 min, reaching (72.7±1.6 mm), compared to the control group (81.5±1.3 mm) ( Table 6).

**Table 6.** The effect of subcutaneous 0.5 ml protoscolex injection on *Candida albicans* yeast phagocytosis.

Concentration	30 min	60 min	90 min	120 min
0.5	72.7±1.6 ^a^	78.6±2.9 ^a,c^	77.9±2.1 ^a,c^	75.6±3.1 ^a,c^
PBs	81.5±1.3 ^b^	82.1±1.4 ^b^	82.4±1.2 ^a^	69.5±2.5 ^a,c^

Different characters indicate the presence of a significant difference (a,b,c) between the parameters at a level < 0.05.

Concerning the effect of antigen injection and infection of mice with antibodies six months after injection, the results showed no significant differences between animals injected with the antigen compared to the control group for all types of antibodies (IgG, IgA, and IgM), as shown in Table 7.

**Table 7.** Effect of protoscolex injection on antibody levels (mg/100 ml) in white mice (Balb/c) 6 months after subcutaneous injection of the antigens.

Concentration	IgG [mg/100 ml]	IgA [mg/100 ml]	IgM [mg/100 ml]
	Mean±SD	Mean±SD	Mean±SD
0.5	1479.3±213 ^a^	358.1±63.3 ^a^	158.1±4.5 ^b^
PBs 0.5	1333.1±183 ^a^	209.9±30.1 ^a^	115.1±23.9 ^b^

Different characters indicate the presence of a significant difference (a,b) between the parameters at a level < 0.05.

Regarding the effect of antigen injection on the percentage of protection against hydatid cysts, Table 8 shows that the percentage of protection against infection reached 50.4%. and the average diameter of hydatid cysts was 3.4±0.4 mm in the group injected with the antigen, compared to the control group, which had an average diameter of 5.4±0.6 mm.

**Table 8.** Effect of protoscolex antigen injection subcutaneous concentrically 0.5 ml on percentage of prevention hydatid cyst injury after 6 months.

Concentration	No. of mice	No. of injured animals	Average of cyst	Percentage of prevention %	Average of cysts diameter
0.5	5	5	1.8±0.4 ^b^	50.4	3.4±0.4 ^b^
PBs	5	5	4.6±1.4 ^a^	0	5.4±0.6 ^a^

Different characters indicate the presence of a significant difference between the parameters at a level of < 0.05.

## Discussion

Table 1 agrees with the findings of Humide *et al.,* ^14^ which recorded a significant increase in IgG and IgA antibodies when injected with protoscolex antigens at a concentration of 0.5 and 0.75, respectively, compared to the control group, whose concentrations reached 1013.3±0.87 and 1385.2±0.87, respectively, for IgG and IgA. This is consistent with the findings of Bdewi *et al.* ^15^ when injected with protoscolex antigens subcutaneously at a concentration of 0.1, which is consistent with the findings of Mahdi *et al.* ^9^ which indicated an increase in the percentage of immunoglobulin type IgG. These results are consistent with those obtained by Humide *et al.* ^14^ and Bdewi *et al.* ^15^ who observed an increase in complement concentration when they injected parasite antigens into the muscle and peritoneum, respectively. Elevated immunoglobulin (IgG, IgA, and IgM) and complement (C3 and C4) levels indicate the activation of immunoglobulins and complements that may contribute to neutralizing or damaging parasitic antigens before or during cyst formation.

As for the reduction of nitroblue tetrazolium dye ( Table 3), which indicates an increase in the dye reduction rate by PMNs cells and an increase in the phagocytosis rate by neutrophils ( Table 6), our results did not indicate any significant differences between the treatments injected with the antigen and the control treatments. This is consistent with the findings of Al Mukhtar ^16^ and Ahmad *et al.* ^17^ This may indicate that protoscolex antigen-induced immune stimulation may focus more on lymphoid pathways (T and B cells) to produce antibodies and not necessarily on activating effector cells such as neutrophils or controlling phagocytic functional enhancement.

While the results in Table 4 indicate significant differences between the experimental treatments and the control treatments, this does not agree with (3). This may be due to differences in the concentrations of antigens used in the experiment or other environmental factors. Table 5, which indicates a significant difference between the antigen-injected groups compared to the control group, is consistent with the findings of Al Mukhtar. ^16^ The results indicated no significant increase in antibody levels 6 months after infection. This may be because hydatid cysts are surrounded by host tissue, which prevents the antigens present within the hydatid cyst from being recognized.

Regarding the extent to which antigen injections affect the ability to prevent or reduce infection, the results showed that the protection achieved by administering antigen doses at a concentration of 0.5 ml resulted in a 50.4% reduction in infection rate compared to the control group. It also resulted in a decrease in the diameter of the hydatid cysts, which reached 3.4±0.4 compared to 5.4±0.6 in the control group, this is consistent with the findings of Humide *et al.* ^14^; Bdewi *et al.* ^15^ This may be due to the ability of the protoscolex antigen to stimulate an immune response. ^18^ and has adsorptive components with the capacity of B and T cells. ^19^ In addition, it shows that injecting this antigen at a concentration of 0.5 ml may lead to a kind of protection or variation in disease progression. This finding is consistent with the idea that prior immune stimulation may reduce the number of parasites that successfully establish themselves or grow.

Despite the important results shown by this study in stimulating the immune response and reducing infection levels, future experiments using other antigens at different protein concentrations are recommended.

## Conclusion

This study demonstrated that the protoscolex antigen possesses significant immunomodulatory activity, stimulating both innate and adaptive immune responses, and reducing the incidence and diameter of hydatid cysts in mice. These results suggest that it could be considered a promising candidate for developing a vaccine against hydatid cyst disease, although the sustainability of the immune response should be improved through the use of adjuvants or a booster dose schedule.

## Ethical approval

This study was conducted in accordance with the principles of Helsinki for medical research on human participants. All participants were informed of the nature and purpose of the study and had the right to withdraw from the study at any time. Informed verbal consent was obtained from the participants, as the study was low-risk and did not involve the collection of personally identifiable information.

All animal procedures were performed according to Russell and Burch’s 3R principle in 1959 ^20^ to ensure the minimum number of animals used, minimize pain and stress, and provide appropriate environmental conditions and care. The study was approved by the Ethics Committee of Fallujah University (Approval No.: UOF.MED.05-240911001).

## Data Availability

Owing to ethical constraints related to participant confidentiality, as stipulated by the Ethics Committee of Fallujah University the raw participant-level data are not publicly available. The study was approved by IRB on the condition that access to the data be restricted to qualified researchers for scientifically and ethically approved purposes. Requests for data access may be submitted to the corresponding author and are subject to approval by the Ethics Committee. Any data sharing will be conducted in de-identified form and governed by a data-use agreement. The ARRIVE checklist for this study has been deposited in Zenodo: ARRIVE Checklist for (Immunological Effects of Protoscolex Antigen Injection on Hydatid Cyst Development in Mice), DOI: https://doi.org/10.5281/zenodo.18160723, licensed under CC0.
