# Supplementary material for: Immunological Effects of Protoscolex Antigen Injection on Hydatid Cyst Development in Mice
Source: F1000Res. 2026 Jan 29;15:132. [Version 1] doi: 10.12688/f1000research.174636.1 (PMC12963828; doi:10.12688/f1000research.174636.1)
Supplement: Peer review history

# Reviewer report: Reviewer response for version 1

**DOI:** 10.5256/f1000research.192555.r458775

**Published:** 2026-03-05

**Authors:** Muthanna Sultan

**Affiliations:** 1 Tikrit University, Tikrit, Iraq

The article looks great  but please add the followings

 1-Materias & Methods

 -Please mention the details about Collection of the hydatid cyst for the injured people.

 -Make sure about the weight of the mice and weight as it supposed to be 20 gm 

 -Also please correct the abbreviation g (gm instead of g)

 2-Discussion 

 The first paragraph of the discussion please add references about I gG & IgM 

 Additional note:

 This article looks great and I Highly recommend it for indexing.

 Thank you!

 Muthanna Sultan Ph.D.

 Biomedical Science / Immunology & microbiology

Is the work clearly and accurately presented and does it cite the current literature?

Yes

If applicable, is the statistical analysis and its interpretation appropriate?

Yes

Are all the source data underlying the results available to ensure full reproducibility?

Yes

Is the study design appropriate and is the work technically sound?

Yes

Are the conclusions drawn adequately supported by the results?

Yes

Are sufficient details of methods and analysis provided to allow replication by others?

Partly

Reviewer Expertise:

Biomedical Science /Immunology & Microbiology

I confirm that I have read this submission and believe that I have an appropriate level of expertise to confirm that it is of an acceptable scientific standard.

# Reviewer report: Reviewer response for version 1

**DOI:** 10.5256/f1000research.192555.r460724

**Published:** 2026-02-27

**Authors:** Noor Hanoush

**Affiliations:** 1 University of Anbar, Ramadi, Iraq

**Background:** Hydatid disease is a widespread disease that affects all human organs.”

**Comment 1:** This statement is overly general and scientifically inaccurate; hydatid disease primarily affects liver and lungs.

**Aims:** This study aimed to evaluate antibodies (IgA, IgG, and IgM), complement proteins (C3 and C4), MIF of PMNs cells

**Comment 2:** The aim is descriptive but lacks a clear hypothesis or research question. protoscolex antigen induces protective immunity and reduces cyst development.

**Introduction:** Hydatid cysts are a common zoonotic disease…”

**Comment 3:** The introduction provides general background but lacks a clear research gap. Add a paragraph explaining why protoscolex antigen vaccination is underexplored and what knowledge gap this study addresses.

**Results**

 -There were significant differences between the treatment and control group.

**Comment 4:** Statistical methods are not described in sufficient detail, and no exact p-values are reported.

**Table 1 –** Antibodies IgG, IgA and IgM antibody level reached…”

**Comment 5:** Units mg/100 ml are unusual for immunoglobulin measurement in mice. Use standard units (μg/mL) and specify ELISA calibration standards.

**Table 2 –** Complement C3 and C4 levels showed a significant decrease…”

**Comment 6: **This contradicts the discussion section claiming complement activation.

**Phagocytosis:** significant decrease in phagocytosis…”

**Comment 7:** This contradicts the hypothesis of immune enhancement.

**Discussion:** Elevated immunoglobulin and complement levels indicate activation…”

**Comment 8:** This contradicts reported complement reduction.

 Correct interpretation and reconcile results. suggested as a promising vaccine candidate…”

**Comment 9: **Overstated claim given weak long-term immunity.

 -Rephrase cautiously and acknowledge limitations.

 Decision: Minor Revision

 The study addresses an important topic and presents preliminary findings; however, methodological weaknesses, statistical limitations, inconsistent interpretation, and language issues significantly reduce scientific rigor.

Is the work clearly and accurately presented and does it cite the current literature?

Partly

If applicable, is the statistical analysis and its interpretation appropriate?

I cannot comment. A qualified statistician is required.

Are all the source data underlying the results available to ensure full reproducibility?

Yes

Is the study design appropriate and is the work technically sound?

Yes

Are the conclusions drawn adequately supported by the results?

Partly

Are sufficient details of methods and analysis provided to allow replication by others?

Yes

Reviewer Expertise:

Immunological, clinical  analysist, biology

I confirm that I have read this submission and believe that I have an appropriate level of expertise to confirm that it is of an acceptable scientific standard.

## References

[1] : Immunological Effects of Protoscolex Antigen Injection on Hydatid Cyst Development in Mice. *F1000Research* .2026;15: 10.12688/f1000research.174636.1 10.12688/f1000research.174636.1 PMC1296382841800218
